# Dynamics of transcriptional enhancers and chromosome topology in gene regulation

**DOI:** 10.1111/dgd.12597

**Published:** 2019-02-19

**Authors:** Moe Yokoshi, Takashi Fukaya

**Affiliations:** ^1^ Institute for Quantitative Biosciences The University of Tokyo Bunkyo‐ku Tokyo Japan; ^2^ Department of Life Sciences Graduate School of Arts and Sciences The University of Tokyo Bunkyo‐ku Tokyo Japan

**Keywords:** enhancers, TADs, transcriptional bursting, transcription hubs, phase separation

## Abstract

Transcriptional enhancers are regulatory DNAs that instruct when and where genes should be transcribed in response to a variety of intrinsic and external signals. They contain a cluster of binding sites for sequence‐specific transcription factors and co‐activators to determine the spatiotemporal specificity of gene activities during development. Enhancers are often positioned in distal locations from their target promoters. In some cases, they work over a million base pairs or more. In the traditional view, enhancers have been thought to stably interact with promoters in a targeted manner. However, quantitative imaging studies provide a far more dynamic picture of enhancer action. Moreover, recent Hi‐C methods suggest that regulatory interactions are dynamically regulated by the higher‐order chromosome topology. In this review, we summarize the emerging findings in the field and propose that assembly of “transcription hubs” in the context of 3D genome structure plays an important role in transcriptional regulation.

## Graphical abstract

Enhancer DNAs play a central role in the spatiotemporal control of gene activities during development. In this review, we summarize the emerging findings in the field and propose that assembly of “transcription hubs” in the context of 3D genome topology is an important feature of enhancer function.

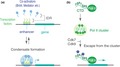

## 1 INTRODUCTION

Most of the developmental and physiological processes rely on precise spatiotemporal patterning of gene expression. Enhancer DNAs play a central role in the control of gene activities in response to developmental timing and environmental cues. They act as a scaffold to recruit sequence‐specific transcription factors and co‐activators, thereby regulating the assembly of active transcriptional machinery at target core promoters. Previous molecular studies have shown that enhancers are separable from core promoter sequences, approximately 80‐bp DNA segments that serve as a docking site of RNA polymerase II (Pol II) (reviewed in Juven‐Gershon, Hsu, Theisen, & Kadonaga, 2008). The first enhancer was originally identified from the genomic DNA of simian DNA tumor virus SV40 by Banerji and Schaffner (Banerji, Rusconi, & Schaffner, 1981). The approximately 200‐bp DNA fragment located upstream of the gene encoding T‐antigen was shown to activate the rabbit *β‐globin* gene from a remote location in an orientation‐independent manner when fused. Just a few years later, the first eukaryotic enhancers were isolated from the intronic regions of mouse *immunoglobulin heavy chain* (*IgH*) and *immunoglobulin kappa* (*IgK*) locus (Banerji, Olson, & Schaffner, 1983; Gillies, Morrison, Oi, & Tonegawa, 1983; Neuberger, 1983; Picard & Schaffner, 1984; Queen & Baltimore, 1983; Queen & Stafford, 1984). Since then, molecular mapping and genome‐wide studies have identified many of key regulatory elements that are critical for the spatiotemporal control of gene activities in development (e.g., Arnold et al., 2013; Kvon et al., 2014). Currently, it is estimated that the human genome contains approximately 400,000 enhancers (ENCODE Project Consortium, 2012), suggesting that a typical human gene is regulated by approximately 20 enhancers. Importantly, many of enhancers are placed distally from their target genes, yet they can specifically communicate with target promoters over a large distance. In some cases, enhancers can act over hundreds of kb or even a few Mb. For example, expression of mouse *Sonic hedgehog* (*Shh*) in developing limb buds is driven by the distal ZRS enhancer located 850 kb away from the promoter region (Lettice et al., 2003; Sagai, Hosoya, Mizushina, Tamura, & Shiroishi, 2005). More strikingly, expression of *Myc* oncogene is regulated by the cluster of enhancers located 1.7 Mb downstream of the promoter (Shi et al., 2013). While chromosome conformation capture (3C) assays and imaging studies suggested that distal enhancers come into physical proximity of target promoters by looping out intervening sequences (e.g., Amano et al., 2009; Dekker, Rippe, Dekker, & Kleckner, 2002), the mechanism and dynamics behind these long‐range interactions still remain as an outstanding mystery. Intriguingly, recent high‐resolution Hi‐C studies have suggested that chromosome topology exerts a significant impact on enhancer–promoter communication and resulting gene expression. Moreover, quantitative imaging methods have provided evidence that enhancers mediate dynamic condensation of transcription factors and co‐activators to drive bursts of de novo transcription, implicating that formation of “transcription hub” is the critical feature of enhancer function. In this review, we summarize recent progress in the field and discuss the emerging new roles of transcriptional enhancers and 3D genome structures in gene regulation.

## 2 THE ROLE OF CHROMOSOME TOPOLOGY IN TRANSCRIPTIONAL REGULATION

**Figure 1:**
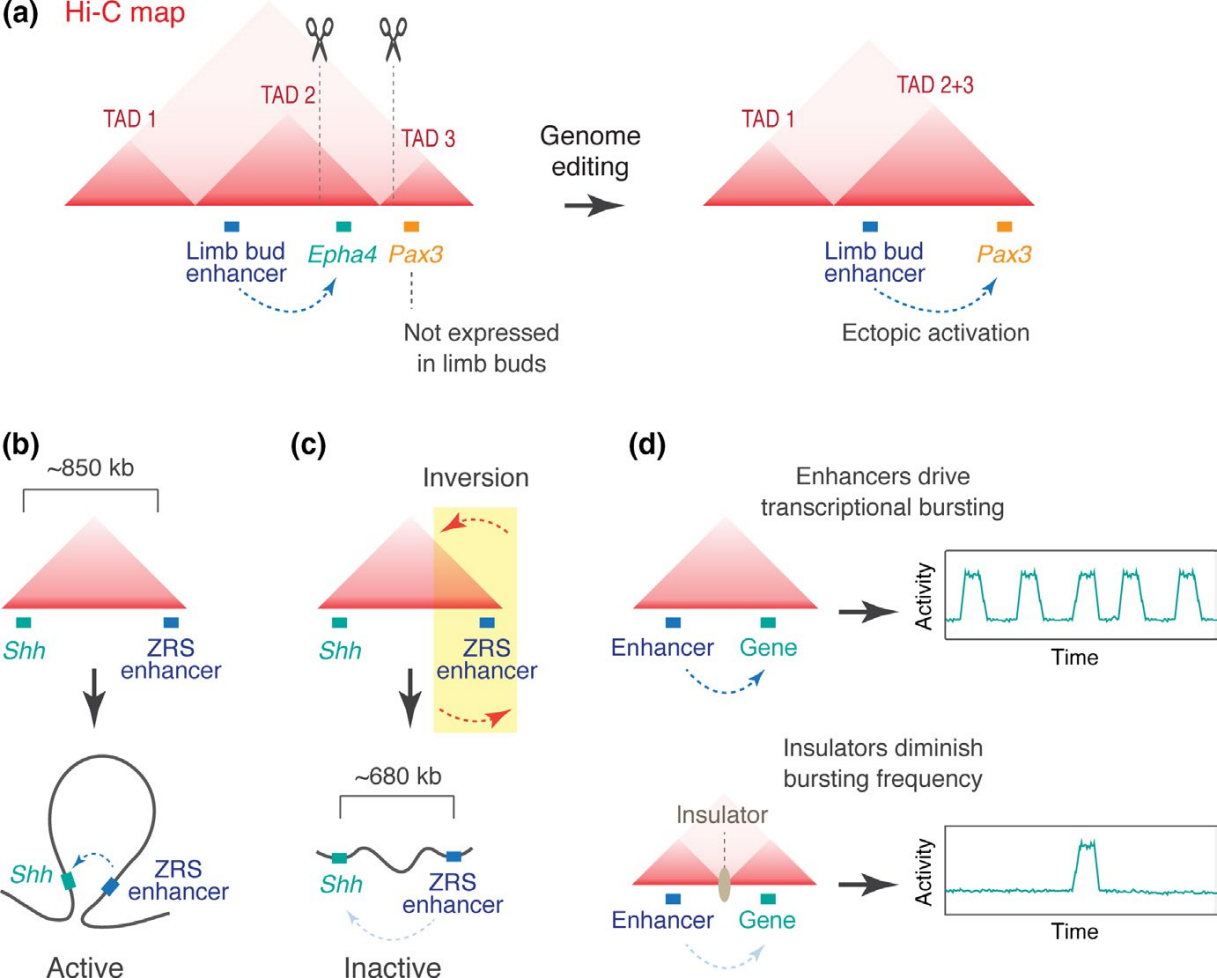
Roles of topologically associating domains (TADs) in the control of enhancer–promoter interaction. (a) Loss of TAD boundary leads to inter‐TAD enhancer–promoter interaction and ectopic gene expression. (b) TAD facilitates intra‐domain interactions. (c) Genome inversion disrupts long‐range enhancer–promoter interaction. (d) Enhancers modulate the frequency of transcriptional bursting (top). Insulator DNAs diminish bursting frequencies (bottom).

Recent progress in 3C technologies and chromatin immunoprecipitation (ChIP) assays has revealed the regulatory landscapes of three‐dimensional genome topology (e.g., ENCODE Project Consortium, 2012, Lieberman‐Aiden et al., 2009). Specifically, recent Hi‐C studies suggested that self‐associating loop domains, or topologically associating domains (TADs), serve as a basic structural unit that consists of higher‐order chromosomal organization (Dixon et al., 2012; Nora et al., 2012). The typical size of TADs is hundreds of kb to Mbs in humans and tens of kb to hundreds of kb in *Drosophila* (Dixon et al., 2012; Ulianov et al., 2016). Regulatory DNAs and their target genes are mostly located within the same topological domain, suggesting that TADs help to ensure the specificity of gene expression by blocking undesirable inter‐TAD contacts. Supporting this view, much evidence has been provided that loss of TAD boundaries causes novel interaction between separate domains, leading to inappropriate inter‐TAD enhancer–promoter communication and ectopic gene expression. For example, in human and mouse, *Epha4* and *Pax3* locus are separated in two neighboring domains, and only the *Epha4* gene is transcriptionally active in limb buds of developing embryos. However, when the TAD boundary was disrupted by genome editing, the *Epha4* enhancer starts to ectopically activate *Pax3* expression, resulting in morphological shortening of the digits (Figure 1a; Lupiáñez et al., 2015). It has also been reported that mutations in boundary elements leads to the activation of proto‐oncogenes such as *TAL1*, a master oncogenic transcription factor in T‐cell acute lymphoblastic leukemia (Hnisz et al., 2016). More recently, many disease‐associated tandem repeats were found to be located in topological boundaries (Sun et al., 2018). Thus, non‐coding mutations in boundary elements are now thought to be a major source of human disease. Overall, these studies support the idea that TADs limit inappropriate inter‐TAD enhancer–promoter interactions to prevent promiscuous transcriptional activation.

In addition, recent studies of mouse *Shh* locus provided evidence that TAD formation also facilitates long‐range enhancer–promoter interaction within a domain (Figure 1b). Expression of *Shh* in the zone of polarizing activity of developing limb buds relies on distal ZRS enhancer positioned approximately 850 kb away from the *Shh* promoter (Lettice et al., 2003; Sagai et al., 2005), both of which are located within a single TAD. Interestingly, when the TAD is disrupted by genome inversion, the distal ZRS enhancer can no longer activate *Shh* expression despite a shorter genomic distance than normal (Figure 1c; Symmons et al., 2016), suggesting that the domain configuration is more critical than the linear distance possibly because TAD brings the distal enhancer into physical proximity of the *Shh* promoter to facilitate their interaction. Recent work using structured‐illumination microscopy (SIM) also concluded that domain configuration optimizes long‐range enhancer–promoter interaction at the *Shh* locus (Williamson, Lettice, Hill, & Bickmore, 2016).

## 3 REGULATORY DYNAMICS WITHIN TADS

While TAD formation seems to be mostly invariant even after differentiation (Rao et al., 2014), intra‐TAD interactions look variable among cell types (Smith, Lajoie, Jain, & Dekker, 2016), implicating that regulatory interactions within TADs are highly dynamic. Previous imaging studies revealed dynamic and stochastic nature of transcription, by demonstrating that transcription generally occurs in bursts in many species including *Dictyostelium*, yeast, *Drosophila*, and mammals (e.g., Bothma et al., 2014; Chubb, Trcek, Shenoy, & Singer, 2006; Larson, Zenklusen, Wu, Chao, & Singer, 2011; Pare et al., 2009; Raj, Peskin, Tranchina, Vargas, & Tyagi, 2006). More recently, it has been shown that enhancers regulate the level of mRNA production by modulating the bursting frequency in time and space during *Drosophila* embryogenesis (Figure 1d, top; Fukaya, Lim, & Levine, 2016). Single‐molecule RNA FISH assay in mammalian *β‐globin* locus also concluded that LCR enhancer changes bursting frequency during erythroid maturation (Bartman, Hsu, Hsiung, Raj, & Blobel, 2016). Furthermore, recent single‐cell RNA‐sequencing method provided transcriptome‐wide evidence that enhancers modulate bursting frequency to achieve cell‐type‐specific gene expression (Larsson et al., 2019), supporting the idea that regulation of transcriptional bursting is a general mechanism of gene control conserved across species. These findings are also consistent with the idea that enhancer–promoter interaction and resulting transcriptional bursting are dynamically regulated within the topological domains during development. Intriguingly, when domain organization was altered by placing an insulator DNA between enhancer and its target promoter, the bursting frequency was significantly diminished (Figure 1d, bottom; Fukaya et al., 2016), suggesting that the occurrence of cell‐type‐specific sub‐TAD structures can also contribute to modulation of bursting frequency.

## 4 MOLECULAR MECHANISM OF TAD FORMATION

It has become clear that TAD boundaries are enriched with the binding sites of a Zinc‐finger DNA‐binding protein CCCTF‐binding factor (CTCF; Rao et al., 2014; Sexton et al., 2012; Nora et al., 2012; Dixon et al., 2012). Originally, CTCF has been reported as an insulator protein that blocks enhancer–promoter interactions when positioned between them (Bell, West, & Felsenfeld, 1999). Recent 3C methods suggested that the enhancer‐blocking activity of CTCF relies on its capability of alternating genome configuration to establish TAD boundaries (reviewed in Ong & Corces, 2014). Whole‐genome ChIP studies revealed that most CTCF binding sites co‐localize with cohesin, a ring‐shaped SMC protein complex (Rubio et al., 2008; Wendt et al., 2008), suggesting that these factors cooperatively regulate genome organization. Intriguingly, CTCF‐binding sites have a sequence directionality, and those at TAD boundaries are typically found to be facing with each other in a convergent orientation (Rao et al., 2014), indicating that the relative position and orientation of CTCF sites are the key determinants of genome organization. The most plausible explanation that summarizes these observations is that cohesin molecules are preferentially recruited to convergent CTCF sites to embrace two separated genomic locations in *cis*, which results in the formation of self‐associating loop domains. Supporting this idea, CRISPR‐inversion of CTCF sites at the *protocadherin* and *β‐globin* locus dramatically changes the domain organization and the profile of enhancer–promoter interaction (Guo et al., 2015). Acute depletion of CTCF or Rad21, a kleisin subunit of the cohesin complex, eliminates essentially all TADs observed with population‐based Hi‐C methods (Nora et al., 2017; Rao et al., 2017; Wutz et al., 2017), highlighting the functional importance of these proteins in TAD formation and maintenance. It has also been reported that cohesin‐loading factor Nipbl and unloading factor Wapl play an important role in this process by balancing the extent to which cohesin molecules are associated with the chromatin during interphase (Gassler et al., 2017; Haarhuis et al., 2017; Schwarzer et al., 2017).

**Figure 2:**
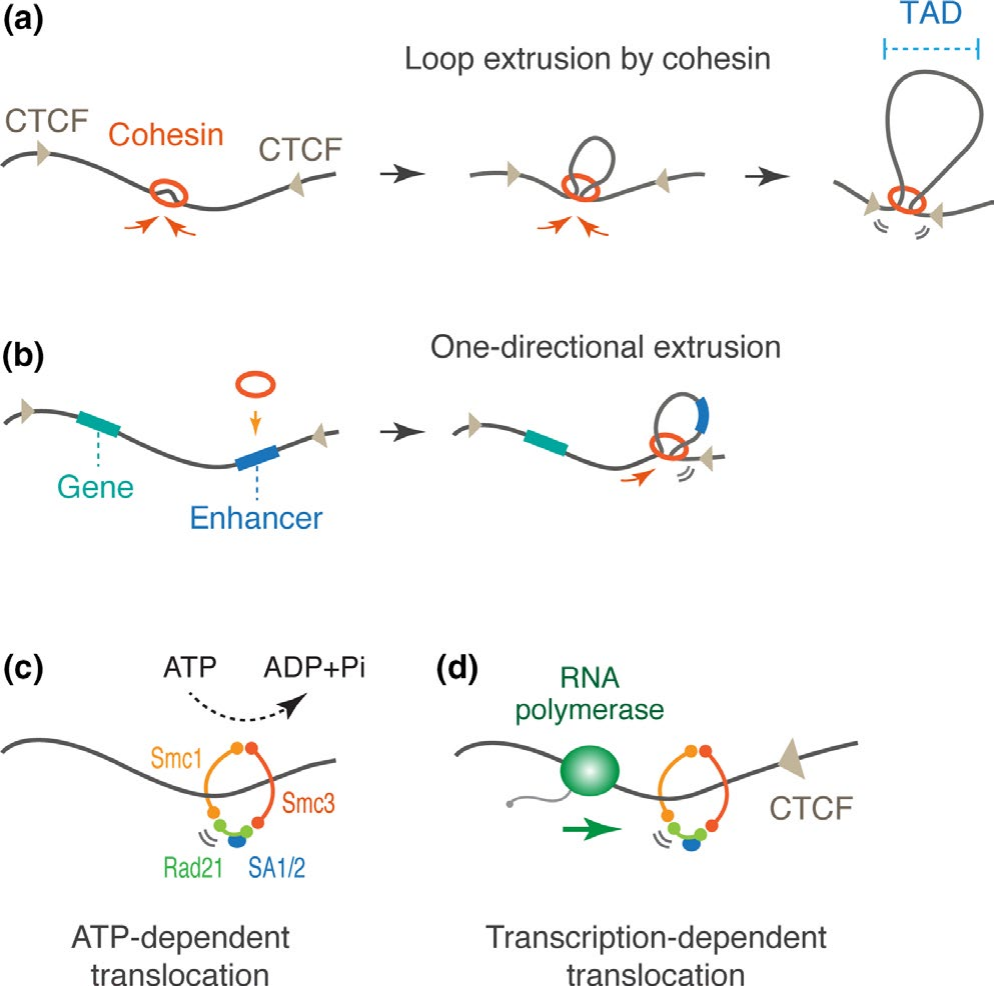
Proposed models for topologically associating domain (TAD) formation. (a) Formation of TADs via loop extrusion. Extruding cohesin stops at the convergent CCCTF‐binding factor (CTCF) sites to from a self‐associating loop domain. (b) Asymmetric loading of cohesin leads to one‐directional loop extrusion. (c) ATP‐dependent translocation model. The cohesin subunits Smc1 and Smc3 have an ATPase activity. (d) Transcription‐dependent translocation model. Elongating RNA polymerase can push over cohesin molecules along chromatin.

Then, how do CTCF and cohesin mediate TAD formation? Recent computational polymer simulations have suggested that cohesin functions as a *cis*‐acting looping factor that progressively extrudes a chromatin fiber to form larger loops until it encounters convergent CTCF sites (Figure 2a; Sanborn et al., 2015; Fudenberg et al., 2016). Indeed, this loop extrusion model seems to be consistent with the experimental data obtained from recent Hi‐C studies in CTCF‐ and cohesin‐depleted cells. For example, in the absence of CTCF, cohesin complex can still bind chromatin to extrude loops but fails to stop at the CTCF sites, leading to the loss of defined TAD boundaries (Nora et al., 2017; Wutz et al., 2017). On the other hand, when cohesin‐unloading factor Wapl is depleted from cells, cohesin complex more stably associates with chromatin during loop extrusion and starts to form extended loops (Gassler et al., 2017; Haarhuis et al., 2017; Wutz et al., 2017), implicating that duration of extruding cohesin is dynamically regulated by Wapl. In contrast, TADs were lost when cohesin‐loading factor Nipbl was depleted since cohesin failed to be recruited to the initiation sites of loop extrusion (Schwarzer et al., 2017).

Interestingly, recent Hi‐C study suggested that Nipbl‐binding sites are often accumulated near the one of convergent CTCF sites. When cohesin is asymmetrically loaded, it immediately reaches one of the CTCF sites and only can extrude loops to the other side. Such one‐directional loop extrusion is implicated to facilitate a boundary element to interact with entire regions within a TAD (Figure 2b; Vian et al., 2018). Super‐enhancers, or large clusters of enhancers, often coincide with asymmetric Nipbl binding sites, suggesting that one‐directional extrusion facilitates long‐range enhancer–promoter interaction and transcriptional activation. However, the functional significance of TAD formation is still under debate since another recent study reported only minor changes in the gene expression profile even after Rad21‐depletion in cultured cell lines (Rao et al., 2017). It might be possible that TADs are more critical in determining the specificity rather than the level of gene activities in the context of developmental processes.

## 5 THE MECHANISM OF COHESIN TRANSLOCATION

Recent single‐molecule imaging studies revealed that condensin, a SMC protein complex that mediates mitotic chromosome assembly, acts as an ATP‐dependent motor that can extrude loops of non‐chromatinized DNA when tested in vitro (Ganji et al., 2018; Terakawa et al., 2017). This observation suggests that cohesin, a related SMC protein complex, also mediates loop extrusion in an ATP‐dependent manner. Supporting this view, recent Hi‐C study reported that ATP depletion leads to loss of TADs in cells, implicating that ATP‐fueled cohesin extrusion, which is presumably catalyzed by Smc1 and Smc3 ATPase subunits of the complex, mediates TAD formation during interphase (Figure 2c; Vian et al., 2018). However, direct experimental evidence of cohesin extrusion has not been obtained yet, and thus, it is still under debate whether cohesin acts as an ATP‐dependent translocation enzyme by itself. Importantly, recent single‐molecule imaging study reported that RNA polymerase can push over cohesin molecules along DNA until it encounters CTCF site in vitro (Figure 2d; Davidson et al., 2016). Consistent with this result, cohesin accumulates at the 3′ ends of convergently transcribed genes in a transcription‐dependent manner in yeast (Glynn et al., 2004; Lengronne et al., 2004). Also, in mammalian cells, formation of “cohesin islands” at 3′ ends of active genes was seen in CTCF/Wapl double‐knockout cells (Busslinger et al., 2017). These results clearly show that transcription can influence translocation of cohesin along chromatin. However, even after global inhibition of transcription by alpha‐amanitin or triptolide, TADs were still formed in developing *Drosophila* embryos (Hug, Grimaldi, Kruse, & Vaquerizas, 2017), suggesting that the transcription‐independent mechanism also supports TAD formation. It might be possible that multiple different mechanisms cooperatively mediate cohesin translocation to shape the interphase chromosome topology. Future biochemical/biophysical studies and single‐cell analysis will provide a concrete molecular explanation for cohesin translocation that underlies 3D genome organization.

## 6 DYNAMICS OF INTERPHASE CHROMOSOME TOPOLOGY

Hi‐C assays using populations of cultured cells showed that TADs are mostly invariant across cell types (Rao et al., 2014; Smith et al., 2016). In contrast, recent single‐cell Hi‐C methods reported that genome topologies are highly variable even among the same cell types (Flyamer et al., 2017; Nagano et al., 2013, 2017; Stevens et al., 2017), suggesting that TADs seen in bulk approaches emerge as a consequence of population averaging of individual unique configurations. Supporting this view, single‐cell imaging of topological domains revealed that the genome can adopt different 3D configurations by anchoring different CTCF/cohesin sites (Bintu et al., 2018). Averaging of individual structures faithfully recapitulates the previously reported population‐based Hi‐C profiles, supporting the idea that seemingly invariant TADs appear as an average of highly dynamic genome configuration. Strikingly, even when Rad21 was depleted by the auxin‐inducible degron system (Natsume, Kiyomitsu, Saga, & Kanemaki, 2016), TAD‐like structures were still seen at the single‐cell level (Bintu et al., 2018). These structures arise by anchoring random genomic locations without any site preference, resulting in loss of TADs in population averaged profiles. Importantly, this gives rise to the possibility that cohesin itself is not required for TAD formation per se. Instead, it is likely that cohesin restricts non‐specific interactions by facilitating the anchoring of specific convergent CTCF sites. Indeed, this model is consistent with the recent studies of somatic homolog pairing in fruit flies. Physical association of homologous chromosomes is thought to be a widespread mechanism in the *Drosophila* genome that can lead to *trans*‐homolog enhancer–promoter communication, or transvection (reviewed in Fukaya & Levine, 2017). It is conceivable that cohesin molecules embrace two homologs as they do for cohesion of sister chromatids during interphase. However, even when Rad21 was depleted from *Drosophila* S2 cells, *trans*‐homolog associations were still maintained (Senaratne, Joyce, Nguyen, & Wu, 2016), suggesting that cohesin‐independent mechanism underlies interactions between homologous chromosomes. Future studies should address the nature of cohesin‐independent genome interactions both *in cis* and *trans*.

## 7 MECHANISM AND FUNCTION OF TRANSCRIPTIONAL CONDENSATES

High‐resolution 3C methods and imaging studies have suggested that transcriptional enhancers can often physically associate with each other (e.g., Allahyar et al., 2018; Beagrie et al., 2017), implying that enhancers have a “sticky” property that mediates their self‐aggregation within a nucleus. Importantly, when cohesin is depleted from cells, enhancer–enhancer contacts start to occur at unusually high frequency (Rao et al., 2017), suggesting that TADs restrict inappropriate aggregation of enhancers during transcriptional activation. Interestingly, recent studies reported that many of transcription factors and co‐activators contain intrinsically disordered regions (IDRs). IDRs are a class of polypeptide segments with a high content of hydrophilic amino acids that can drive liquid–liquid phase‐separation via multivalent interaction of IDR‐containing proteins. In recent years, it is becoming increasingly clear that phase‐separation plays a fundamentally important role in widespread biological processes including assembly of cytoplasmic RNA granules (Molliex et al., 2015), nuclear paraspeckles (Yamazaki et al., 2018), nucleoli (Feric et al., 2016), and heterochromatin (Larson et al., 2017; Strom et al., 2017). Notably, recent imaging and biochemical studies have provided evidence that IDRs in the transcription apparatus drive phase‐separation or condensate formation that may serve as a “hub” for transcriptional activation (Boija et al., 2018; Cho et al., 2018; Chong et al., 2018; Sabari et al., 2018; Shin et al., 2018).

**Figure 3:**
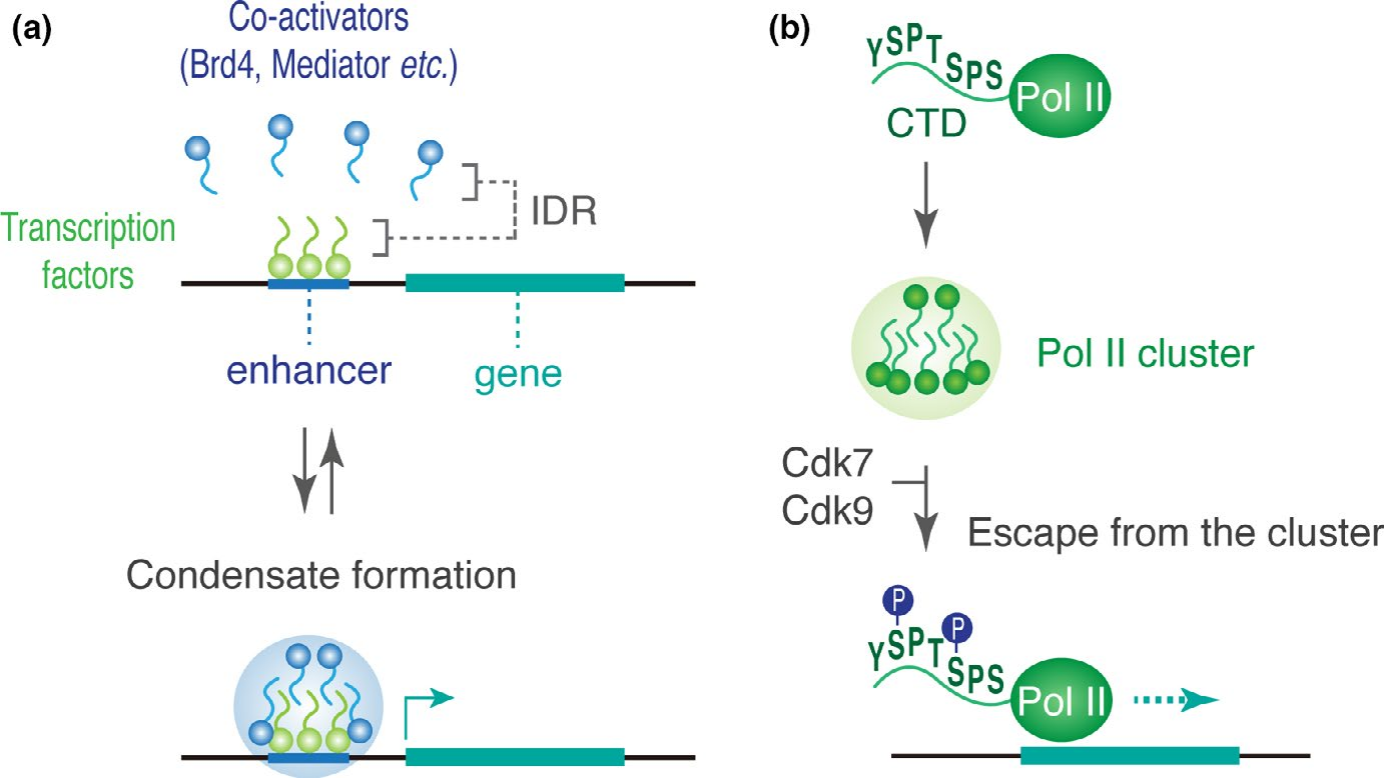
Gene control via dynamic condensation of transcriptional apparatus. (a) Many of transcription factors and co‐activators contain intrinsically disordered regions (IDRs) that can induce formation of phase‐separated droplets in cells. (b) Heptapeptide repeats in C‐terminal domain (CTD) can mediate dynamic clustering of Pol II molecules in cells. Upon phosphorylation of Ser5 (catalyzed by Cdk7) and Ser2 (catalyzed by Cdk9), Pol II initiates productive transcription elongation.

Brd4, a member of the bromodomain protein family, is a major transcriptional co‐activator that binds to acetylated histones (e.g., H3K27ac) and transcription factors at enhancers (Chapuy et al., 2013; Dey, Chitsaz, Abbasi, Misteli, & Ozato, 2003). The C‐terminal domain of Brd4 contains a characteristic disordered region that can drive dynamic condensation via phase‐separation in vivo (Sabari et al., 2018). Similarly, Med11, a key subunit of the Mediator co‐activator complex, is also capable of inducing phase‐separation through its conserved C‐terminal IDR. When cells were treated with 1,6‐hexanediol that perturbs weak hydrophobic protein interactions (Patel, Belmont, Sante, & Rexach, 2007), the level of Brd4 and Med11 association at enhancers was diminished, which results in concomitant reduction of transcription activities of target genes (Sabari et al., 2018). More recently, it has been shown that the activation domain of well‐characterized transcription factors such as mouse Oct4 and yeast GCN4 can drive the formation of phase‐separated droplets that colocalize with Mediator droplets (Boija et al., 2018). Also, in early *Drosophila* embryos, clustering of key transcription factors such as Zelda, Bicoid, and Ultrabithorax has been reported so far (Dufourt et al., 2018; Mir et al., 2017; Tsai et al., 2017). Thus, it can be possible that enhancers act as a scaffold where transcription factors and co‐activators dynamically accumulate for subsequent recruitment of Pol II molecules to target genes (Figure 3a).

Consistent with this idea, recent super‐resolution live imaging studies revealed that not only the Mediator complex but also Pol II forms dynamic condensates in mammalian cells (Cho et al., 2016, 2018; Cisse et al., 2013). While most of these Pol II condensates are short‐lived, they also form large stable clusters within a nucleus in mouse ES cells. When phosphorylation of Ser2 at the C‐terminal domain (CTD) of Pol II was inhibited by 5,6‐dichlorobenzimidazone‐1‐B‐D‐ribofuranoside (DRB), stable Pol II clustering was lost. Since the Ser2 phosphorylation by Cdk9 is essential for Pol II to initiate productive transcriptional elongation (Rahl et al., 2010), it is likely that the stable clusters are formed as a consequence of active transcription. In contrast, the short‐lived Pol II clusters seem to be condensates of unphosphorylated complexes that may contribute to consecutive initiation and subsequent elongation during transcriptional bursting (Cho et al., 2016). Consistent with this model, a recent biochemical study reported that YSPTSPS heptapeptide repeats in the Pol II CTD induce formation of phase‐separated droplets that can facilitate local accumulation of Pol II molecules both in vitro and in vivo (Boehning et al., 2018). Importantly, upon phosphorylation, Pol II escapes from the droplets to start productive elongation (Kwon et al., 2013), suggesting that the dynamic assembly and disassembly processes of short‐lived clusters are regulated by the phosphorylation state of the Pol II CTD (Figure 3b). Intriguingly, the elongation factor p‐TEFb, a complex of Cyclin T1 and Cdk9, is also implicated to undergo phase‐separation to facilitate the Ser2 phosphorylation during transcriptional activation (Lu et al., 2018). Overall, these recent findings are consistent with the model in which dynamic condensation of transcription factors, co‐activators, elongation factors and Pol II complexes contributes to gene expression by producing a microenvironment that compartmentalizes transcription reactions within the nucleus.

## 8 FUNCTIONAL INTERPLAY BETWEEN TRANSCRIPTION HUBS AND CHROMOSOME TOPOLOGY

In the traditional view, enhancers are thought to interact with promoters through formation of stable loops in a targeted manner. According to this model, it is expected that a single enhancer can activate only one target promoter at a given time. However, recent live imaging studies revealed that a shared enhancer can co‐activate multiple linked genes *in cis* (Fukaya et al., 2016). More recently, it has also been reported that a single enhancer can drive co‐activation of two reporters across homologous chromosomes (Lim, Heist, Levine, & Fukaya, 2018). These observations appear to be consistent with the emerging model where enhancers activate target promoters through formation of “transcription hubs,” rather than mutually exclusive looping interactions.

**Figure 4:**
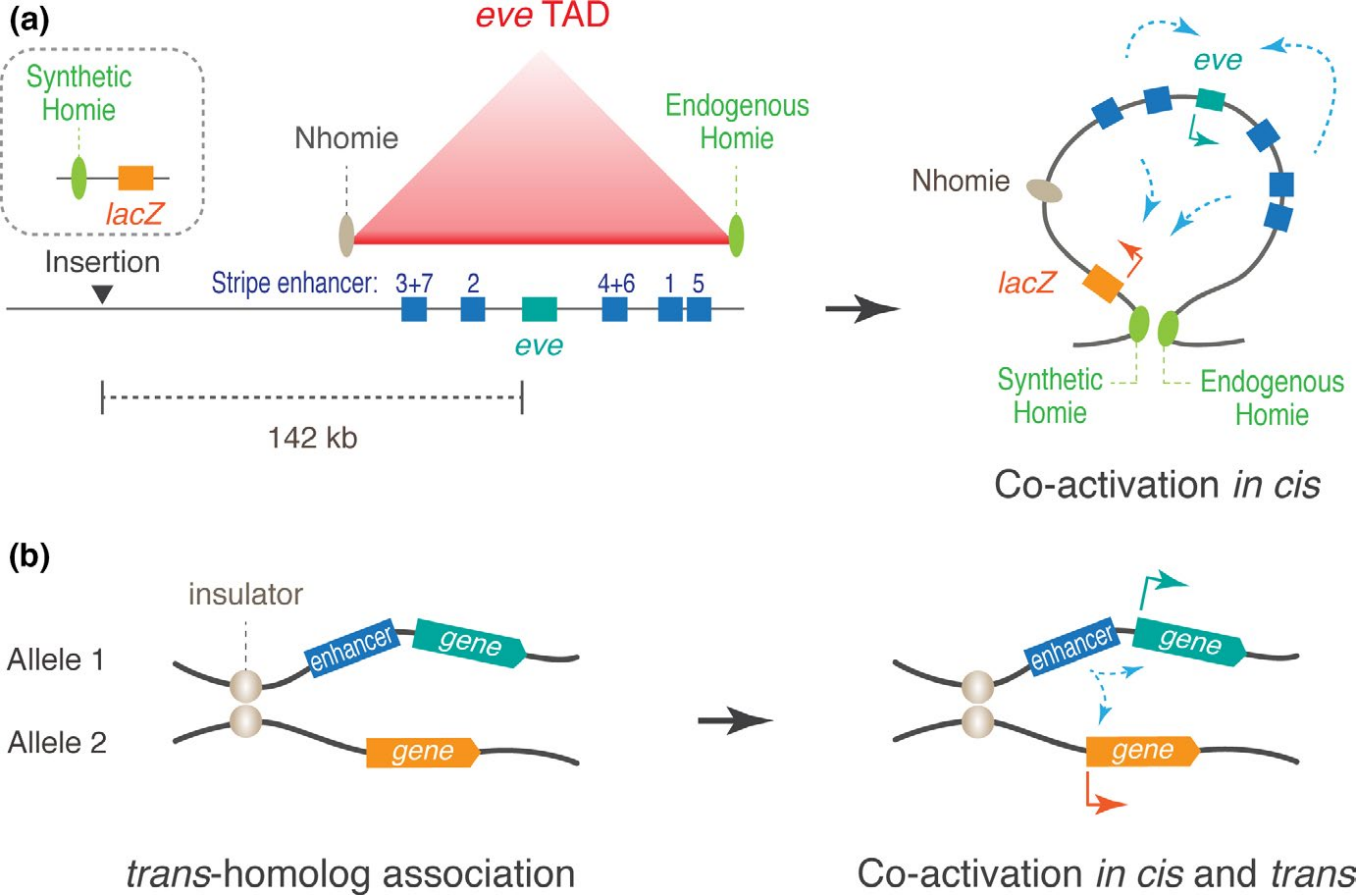
Co‐activation of multiple promoters by shared enhancers. (a) Organization of the endogenous *eve* locus in *Drosophila*. Insertion of synthetic Homie insulator‐*lacZ* construct induces re‐organization of genome configuration via Homie‐Homie insulator pairing, leading to co‐activation of *eve* and *lacZ* by endogenous stripe enhancers. (b) Pairing of insulators facilitates stable homolog association and co‐activation of two reporters by a shared enhancer

While it appears that enhancers are intrinsically capable of co‐activating multiple promoters when tested (Fukaya et al., 2016), specificity of regulatory interactions seems to be tightly regulated by the local genome configuration to prevent promiscuous transcriptional activation. For example, in the *Drosophila even‐skipped* (*eve*) locus, stripe enhancers and *eve* transcription unit are all embedded in a single TAD that is bordered by two insulators, Homie and Nhomie (Figure 4a, left; Fujioka, Sun, & Jaynes, 2013; Fujioka, Wu, & Jaynes, 2009; Cubenas‐Potts et al., 2017). When a synthetic enhancer‐less *lacZ* reporter was placed in a remote location outside of the *eve* TAD, the stripe enhancers do not activate *lacZ* (Fujioka et al., 2009). However, when re‐organization of genome structure was induced by using the pairing of endogenous Homie and synthetic Homie, the stripe enhancers start to co‐activate both the endogenous *eve* and synthetic *lacZ* simultaneously (Figure 4a, right; Chen et al., 2018). Similarly, in early fly embryos, *trans*‐homolog co‐activation occurs only when stable association of homologous chromosomes was induced by pairing of insulators (Figure 4b; Lim et al., 2018; Fujioka, Mistry, Schedl, & Jaynes, 2016), indicating that genome configurations significantly change the range that enhancers can act. Importantly, when co‐activation happens, two promoters start to compete with each other for shared pool of the transcription machineries (Chen et al., 2018; Lim et al., 2018), again supporting the occurrence of transcription hubs during gene activation.

## 9 FUTURE PERSPECTIVES

While recent live‐imaging methods have suggested that the formation of transcription hub is an important feature of enhancer function (Chen et al., 2018; Fukaya et al., 2016; Lim et al., 2018), it still remains unclear how enhancers produce such a nuclear microenvironment during transcriptional activation. Since the current evidence of phase‐separation model largely stems from the analysis of super‐enhancers (Boija et al., 2018; Cho et al., 2018; Sabari et al., 2018), it is yet to be determined whether canonical enhancers drive transcription in an IDR‐dependent manner as well. It might be possible that phase‐separation is not a prerequisite for the enhancer function in general, but plays an auxiliary role to help the efficiency of transcription by increasing the local concentration of effector proteins at specific genomic locations. In other words, phase‐separation may contribute to increase the size of transcription hub, but the hub formation itself may not entirely rely on the phase‐separation mechanism. Cleary, future functional studies are needed to fully elucidate the role of IDRs and the mechanism of hub formation. Another major challenge in the field is to define the role of topological domains in the control of enhancer–promoter communication. While a number of genetic studies have shown that loss of TAD boundaries significantly impacts regulatory interactions and spatial patterning of gene activities, a recent genome‐wide method reported that expression profiles are largely unaffected even after Rad21‐depletion in cultured cells (Rao et al., 2017). To reconcile this controversy, it is key to develop a new experimental framework that combines single‐cell imaging methods and genetic approaches to directly visualize the role of topological domains in the context of animal development. The advent of quantitative live‐imaging and genome editing methods has a strong potential to unravel the functional interplay between transcriptional enhancers and chromosome topology, and should greatly augment our current capacity to superimpose whole‐genome regulatory landscapes onto the enhancer dynamics in gene regulation.
